# Rice Lesion Mimic Mutants (LMM): The Current Understanding of Genetic Mutations in the Failure of ROS Scavenging during Lesion Formation

**DOI:** 10.3390/plants10081598

**Published:** 2021-08-04

**Authors:** Sang Gu Kang, Kyung Eun Lee, Mahendra Singh, Pradeep Kumar, Mohammad Nurul Matin

**Affiliations:** 1 Department of Biotechnology, Institute of Biotechnology, Yeungnam University, Gyeongsan 38541, Gyeongbuk, Korea; 2 Department of Forestry, North Eastern Regional Institute of Science and Technology, Nirjuli 791109, Arunachal Pradesh, India; 3 Department of Genetic Engineering and Biotechnology, University of Rajshahi, Rajshahi 6205, Bangladesh

**Keywords:** lesion mimic mutant, spotted leaf, programmed cell death, *Oryza sativa*, hypersensitive response

## Abstract

Rice lesion mimic mutants (LMMs) form spontaneous lesions on the leaves during vegetative growth without pathogenic infections. The rice LMM group includes various mutants, including *spotted leaf* mutants, *brown leaf* mutants, *white-stripe leaf* mutants, and other lesion-phenotypic mutants. These LMM mutants exhibit a common phenotype of lesions on the leaves linked to chloroplast destruction caused by the eruption of reactive oxygen species (ROS) in the photosynthesis process. This process instigates the hypersensitive response (HR) and programmed cell death (PCD), resulting in lesion formation. The reasons for lesion formation have been studied extensively in terms of genetics and molecular biology to understand the pathogen and stress responses. In rice, the lesion phenotypes of most rice LMMs are inherited according to the Mendelian principles of inheritance, which remain in the subsequent generations. These rice LMM genetic traits have highly developed innate self-defense mechanisms. Thus, although rice LMM plants have undesirable agronomic traits, the genetic principles of LMM phenotypes can be used to obtain high grain yields by deciphering the efficiency of photosynthesis, disease resistance, and environmental stress responses. From these ailing rice LMM plants, rice geneticists have discovered novel proteins and physiological causes of ROS in photosynthesis and defense mechanisms. This review discusses recent studies on rice LMMs for the Mendelian inheritances, molecular genetic mapping, and the genetic definition of each mutant gene.

## 1. Introduction

Rice (*Oryza sativa* L.) is a staple food for more than one-third of the world’s population. High-yield rice is needed to meet the demand for the rapidly increasing global population, which is proposed by the United Nations to reach 9.3 billion and 10.1 billion in 2050 and 2100, respectively On the other hand, rice diseases are constantly posing a major threat to meeting the essential foods demand for the global population. For instance, the rice blast disease caused by the fungus *Magnaporthe oryzae* results in a major loss of net rice production [1]. Plants respond to pathogen attacks and develop complex signaling and defense mechanisms to protect themselves [2,3]. 

**Figure 1:**
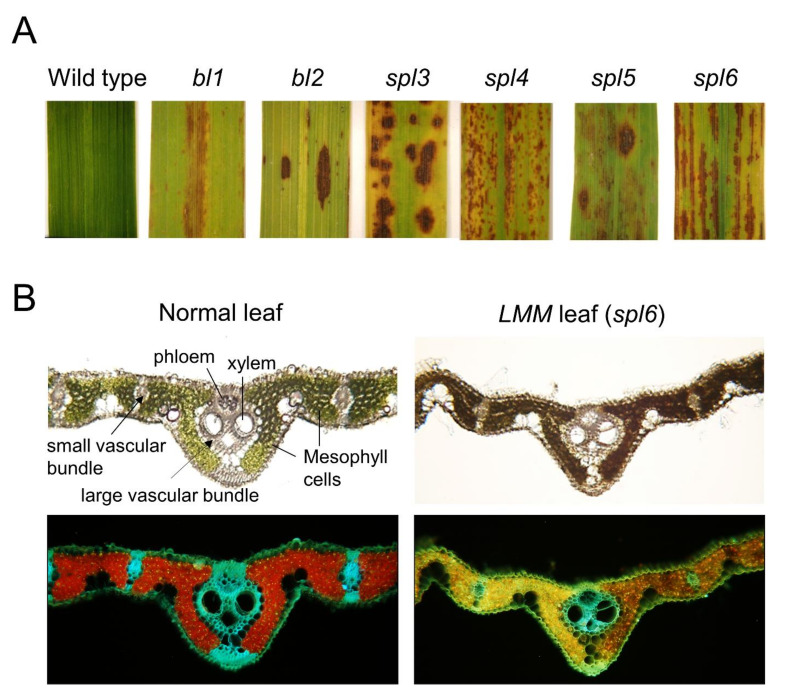
Phenotypes of spot formation on the leaf blade of LMM mutants; *bl1, bl2, spl3, spl4, spl5,* and *spl6*. (**A**) Leaf shape and spot patterns. In each LMM mutant, the left leaves show symptoms of lesion spots. Apparent differences regarding the structure, color, arrangement, and severity of spots at the mature stage are identified. In *spl6*, small spots joined together to form longitudinal lines, leaving non-spotted areas on the midrib. (**B**) Mid-leaf cross-section of wild type and *spl6* mutant under fluorescence optical microscopy observed under UV light at 488 nm. The clear red color of chloroplasts in mesophyll cells was observed as active photosynthesis in the wild-type leaf. The brown and yellow color of mesophyll cells exhibit degraded chloroplasts, resulting in inactive photosynthesis in the mesophyll cells of *spl6* leaves (LMM).

One of the most efficient and immediate resistance reactions is the hypersensitive response (HR), which is characterized by the rapid death of plant cells directly in contact with, or close to the pathogens [4,5,6]. Plants regulate the signaling pathways triggered by HR that leads to cellular programmed cell death (PCD), including in response to pathogen infections. On the other hand, some lesions of PCD appear spontaneously on the leaf surfaces in the absence of a pathogenic infection. These phenotypes are similar to pathogen inducible phenotypes and are called lesion mimic mutants (LMMs) [7,8] (Figure 1).

The phenotypic mutants of LMMs are commonly found in most plant species, including *Arabidopsis* sp. [9,10,11,12], rice [13,14,15,16,17,18,19,20,21,22,23,24,25,26], maize [7,26,27,28,29], barley [30,31,32,33], soybean [34], wheat [35], potato [36], and cotton [37]. The LMMs of rice have been well documented in genetic research, including *spotted leaf* mutants, *brown leaf* mutants, *white-stripe leaf* mutants, and other lesion phenotypic mutants (Table 1). On the other hand, as most LMM plants have a significantly lower grain yield, genetic studies of LMMs have been conducted to understand their negative effects on agronomic traits. The genetic principles of LMMs can be used to understand the molecular network governing plant defense, immunity, and vegetative growth [15]. Moreover, the achievements of molecular genetic studies for LMMs contribute to the utilization of crop breeding.

This paper describes the genetic and molecular functions of mutants with lesion mimic phenotypes in rice and their involvement in other cellular activities. In addition, this paper proposes the application of precious rice gene resources to be used in breeding programs to develop disease resistance and higher yield in rice.

## 2. Inheritance and Genetic Definition of Rice LMMs

In rice genetics, the *spotted leaf* (*spl*) trait of rice LMM has been well documented [38,39] (Figure 1). These *spl* genetic traits, including *spl1, spl3 spl4, spl5, spl6, spl7*, and *spl11*, show a typical recessive genetic inheritance [13,14,18,19,20,21,22,39], whereas the *SPL18* gene is found as the dominant inheritance [22]. Infrequently, *white lesion leaf* mutants, the *white-stripe leaves and panicles 1* (*wspl*) and the *white-lesion mimic leaf1* (*wlml1*) are determined by recessive alleles [40,41]. In addition, light-induced rice LMMs, such as the *light-induced lesion mimic mutant 1* (*LIL1*), are governed by a semi-dominant allelic gene [17]. Therefore, most rice LMM traits are frequently inherited as recessive genetic linked phenotypes.

As the mutant traits reflect the function of the genes, the genetic definition of the rice LMMs has been studied intensively in plant molecular genetics. In particular, the complete DNA sequencing of *Oryza sativa* performed by the International Rice Genome Sequencing Project (IRGSP) essentially helped to decipher the molecular genetics of rice [42]. Moreover, the defined genes of rice LMM traits have been made available to the public since the integration of Rice Annotation Project Data Bases (RAP-DB) in the IRGSP and the MSU Rice Genome Annotation Project [43,44] (Table 1). Science communities, specifically plant geneticists, have been praised for their contributions to building the respective rice genome databases.

Rice lesion mutants have been well documented in genetics and physiology [45,46]. Recently, numbers of the genes caused by the rice LMM phenotypes have been identified using the map-based gene cloning technique in terms of forwarding genetic analysis (Table 1). These genetic analyses are quite accurate because the map-based gene cloning is achieved by simple-sequence repeat (SSR) markers assisting in gene mapping by polymerase chain reaction (PCR) methods based on the Mendelian genetic segregation [47]. 

In the current molecular genetics of rice LMM research, a candidate locus of the phenotypic trait is mapped and cloned for the corresponding gene and then used for genetic complementation analysis; as a result, the genetic definitions of rice phenotypic LMM mutant traits have been defined accurately. Moreover, the Mendel’s dominant and recessive genetic laws are well defined based on the achievements of the molecular genetics of rice LMM studies to date. Therefore, many phenotypic traits of rice LMMs following the Mendelian segregation have been elucidated from their main causal DNA sequences as genes [48,49,50,51]. Table 1 summarizes the characteristics of the cloned rice LLM genes with their respective phenotypes. 

**Table 1:** Identified LMM genes with their gene products, phenotypes, and names in rice.

Gene Mutants	Gene Locus RAP ID, MSU ID	RAP-DB Gene Symbols	CGSNL Gene Symbols	CGSNL Gene Name	Oryzabase Gene Symbol Synonyms	Oryzabase Gene Name Synonyms	Gene Product (Protein)	ProgramMed Cell Death (PCD) Phenotype	Trait Class and Mechanisms Involve	Reference
*bbs1*	Os03g0364400 LOC_Os03g24930	OsBBS1, OsRLCK109	BBS1	BILATERAL BLADE SENESCENCE 1	OsRLCK109, RLCK109, OsBBS1, OSBBS1/OsRLCK109	Receptor-like cytoplasmic kinase 109, bilateral blade senescence 1	Receptor-like cytoplasmic kinase	Dark brown lesions in leaves; Regulates cell death and defense responses	Disease resistance, Receptor-like cytoplasmic kinase 109 regulates cell death	[16,50]
*cea62*	Os02g0110200, LOC_Os02g02000.1	HPL3, OsHPL3, CYP74B2	HPL3	HYDROPEROXIDE LYASE 3	OsHPL3, CYP74B2, OsCYP74B2		Fatty acid 13-hydroperoxide lyase	Brown lesion spot over the entire leaf surface	Disease resistance, insect resistance, viral disease resistance; Due to constitutive induction of JA signaling	[51,52]
*csfl6*	Os08g0160500 LOC_Os08g06380.1	CSLF6, OsCslF6, OsCSLF6	CSLF6	CELLULOSE SYNTHASE LIKE F6	OsCslF6, OsCSLF6, OsCLD3, CLD3		MLG (mixed-linkage glucan) synthase	Spontaneous, discrete, necrotic lesions in flag and old leaves	Due to a decrease in mixed-linkage glucan contents	[53]
*lil1*	Os07g0488400 LOC_Os07g30510	LIL1	LIL1	LIGHT-INDUCED LESION MIMIC MUTANT 1		Light-induced lesion mimic mutant 1	Cysteine-rich receptor-like kinase (CRK),	Light-induced, small, rust-red lesions on the leaf; accumulation of ROS; PCD	LMM, Disease resistance; Putative cysteine-rich receptor-like kinase (CRK) casual light-induced LMM	[17]
*llm1*	Os04g0610800 LOC_Os04g52130.1		RLIN1	RICE LESION INITIATION 1	CPO, CPOX, LLM1, HEMF	Coproporphyrinogen III oxidase, leaf lesion mimic mutant	Coproporphyrinogen III oxidase	Showed programmed cell death and accumulated ROS	LMM, Stress tolerance; Chloroplast damage in lesion formation in rice	[54]
*spl3*	Os03g0160100	OsSPL3, LCRN1	SPL3, OsEDR1 ACDR1	SPOTTED LEAF3 (SPL3)	OsEDR1, OsACDR1, EDR1	Tolerance and resis- tance, lesi- on mimic,	Mitogen-activated protein kinase kinase kinase (MAPKKK),	lesion spots on leaves at the late tiller- ing stage	caused direct- ly by excess amounts of H_2_O_2_ accumulation	[55]
*lms*	Os02g0639000	LMS, OsLMS	LMS	LESION MIMIC AND SENESCENCE	OsLMS		Double-stranded RNA binding domain (dsRBD) containing protein	Reddish-brown lesions on leaves and rapid senescence after flowering	Failure of regulating the stress responses	[56]
*lsd1*	Os08g0159500 LOC_Os03g43840.1	OsLOL1, OsLSD1			OsLSD1, OsLOL1, OsLSD1.1, LSD1.1, LSD1	Zinc finger protein LSD1, LESION SIMULATING DISEASE1.1,	C2C2-type zinc finger protein	Regulates PCD and callus differentiation	Negative regulator of PCD and hypersensitive response (HR)	[57]
*mlo*	Os06g0486300	OsMLO2	MLO_	POWDERY-MILDEW-RESISTANCE GENE O_	OsMLO6, OsMLO1, OsMlo1, Mlo, OsMlo-1, Mlo1,	Powdery-mildew-resistance gene O6,	MLO-like protein 1	Modulating defense responses and cell death	Involved in modulation of pathogen defense and cell death. Ca^2+^-dependent calmodulin-binding	[58]
*Sl*	Os12g0268000 LOC_Os01g50520.1	SEKIGUCHI LESION	OsSL	SEKIGUCHI LESION	OsSL, spl1, CYP71P1, OsLLM1	Sekiguchi lesion, cytochrome P450 71P1, tryptamine 5-hydroxylase, large lesion mimic 1	Cytochrome P450 monooxygenase	Spotted lesions on leaves	Disease resistance, Cytochrome P450 monooxygenase function	[18,59]
*spl4*	Os06g0130000 LOC_Os06g03940	LMR, SPL4	LRD6-6	LESION RESEMBLING DISEASE 6-6	LMR, LRD6-6	Lesion resembling disease 6-6 (LRD6-6)	AAA-type ATPase, Plant spastin	Lesion formation and also affects leaf senescence in rice	LMM, disease resistance, endosomes-mediated vesicular trafficking	[60,61,62,63]
*spl5*	Os07g0203700 LOC_Os02g04950.1		SPL5	SPOTTED LEAF 5	spl5-1	Spotted leaf5	Putative splicing factor 3b subunit 3 (SF3b3) CPSF A subunit region	Small reddish-brown spots scattering over the whole surface of leaves	LMM, splicing factor regulates gene expression,	[49,64]
*spl7*	Os05g0530400 LOC_Os04g48030.1		SPL7,	SPOTTED LEAF 7	spl7, HSFA4D	Spotted lea , spotted leaf-7, heat stress transcription factor Spl7	Heat stress transcription factor	Small, reddish-brown lesions over the whole surface of leaves	LMM, Balancing ROS during biotic and abiotic stress	[14,25]
*spl11*	Os12g0570000 LOC_Os01g60860.1		SPL11	SPOTTED LEAF 11	spl11, spl11*, OsPUB11, PUB11	Spotted leaf11; plant U-box-containing protein 11	Protein with E3 ubiquitin ligase activity; Armadillo-like helical domain-containing protein	Chlorosis and spotted lesions on leaves in LD conditions Red spots distribute on leaf	LMM, disease resistance, E3 ligase negatively regulates PCD	[45]
*spl18*	Os10g0195600		SPL18	SPOTTED LEAF 18	Spl18, OsAT1	Spotted leaf 18	Acyltransferase protein	Formation of necrotic lesion on the leaf. OsAT1 transferase protein	LMM, disease resistance, Hypersensitive reaction in tobacco	[22]
*spl28*	Os01g0703600 LOC_Os01g25110	SPL28	SPL28	SPOTTED LEAF 28			Clathrin-associated adaptor protein complex 1 medium subunit mu1 (AP1M1),	Spotted lesions on leaves and early senescence	Disease resistance, regulation of vesicular trafficking	[48]
*spl30*	Os12g0566300 LOC_Os12g37870	OsACL-A2, SPL30	ACLA2	ATP-CITRATE LYASE A2	SPL30, OsACL-A2, ACL-A2	Spotted leaf 30, ATP-citrate lyase A2	Subunit A of the heteromeric ATP-citrate lyase	Negative regulation of cell death, disease resistance	LMM accumulates ROS and degrades nuclear deoxyribonucleic acids	[65]
*spl32*	Os07g0658400 LOC_Os07g46460.1	OsGLU, OsFd-GOGAT, Fd-GOGAT	ABC1	ABNORMAL CYTOKININ RESPONSE 1	OsABC1, SPL32	Ferredoxin glutamate synthase-spotted leaf 32	Ferredoxin-dependent glutamate synthase	The *spl32* plants displayed early leaf senescence	LMM, decreased chlorophyll, upregulate superoxide dismutase	[66]
*spl33*	Os01g0116600 *LOC_Os01g02720*	SPL33	SPL33	SPOTTED LEAF 33	LMM5.1, SPL33/LMM5.1	Spotted leaf 33, lesion mimic mutant 5.1	Eukaryotic translation elongation factor 1 alpha (eEF1A)-like protein	Negative regulation of cell death, Defense response; death and early leaf senescence	LMM, consisting of a nonfunctional zinc finger domain and three functional EF-Tu domains	[67]
*spl35*	Os03g0205000 *LOC_Os03g10750*	OsSPL35		SPOTTED LEAF 35		Spotted leaf 35	CUE (coupling of ubiquitin conjugation to ER degradation) domain-containing protein	Induce cell death resulting lesion mimic mutant and enhanced disease resistance to fungal and bacterial pathogens	LMM, Decreased chlorophyll content, accumulation of H_2_O_2,_ and upregulated defense-related genes	[68]
*spl40*	Os05g0312000	SPL40	SPL40	SPOTTED-LEAF 40	OsMed5_1, Med5_1	Mediator 5_1, spotted-leaf 40		Cell death around the lesion and burst of ROS	Disease resistance; Bacterial blight resistance	[69]
*wsp1*	Os04g0601800 LOC_Os05g0482400	WSP1	WSP1	WHITE-STRIPE LEAVES AND PANICLES 1	OsWSP1, OsMORF2b, MORF2b	White-stripe leaves and panicles 1, multiple organellar RNA editing factor 2b	Multiple organellar RNA editing factor (MORF) family protein	Brown spots and white lesion mimic spots on the tip and leaves	Stress tolerance; brown spots and white lesion mimic spots on the tip of the leaves	[70]

CGSNL: The Committee on Gene Symbolization, Nomenclature, and Linkage [71]; Oryzabases: (https://shigen.nig.ac.jp/rice/oryzabase/, last accessed on 3 August 2021); RAP: The Rice Annotation Project Database (RAP-DB), (https://rapdb.dna.affrc.go.jp/, last accessed on 3 August 2021); MSU: The Michigan State University Rice Genome Annotation Project, (http://rice.plantbiology.msu.edu/, last accessed on 3 August 2021).

The rice *Sekiguchi lesion* (*SL*) mutant was identified on the Os12g0268000 locus as involved in cell death and disease resistance [18,72]. The *SL* gene encoded a tryptamine 5-hydroxylase of cytochrome P450 monooxygenase family protein that catalyzes the conversion of tryptamine to serotonin [73]. The SL protein functions as NADPH-dependent tryptamine 5-hydroxylase to remove oxygen. The *SL* phenotype showed an increase light-dependent tryptophan decarboxylase and monoamine oxidase activities upon infection with *Magnaporthe grisea* [73]. 

The *SL* gene expression was induced by the chitin elicitor and by infection with a rice blast disease pathogen, *M. grisea* [59,73]. In the *SL* mutant studies, the endogenous plant serotonin-induced defense gene expression plays a vital role in the plant’s innate immunity. Tian et al. reported that a loss of the functional mutant in the *SL* allele of Minghui 86 resulted in higher levels of defense hormones, such as salicylic acid and jasmonic acid, and suggested to enhance the pathogen triggered immunity responses [74].

The rice *spotted leaf 3* (*spl3*) mutant was induced by gamma rays and manifested as lesion spots on leaves at the late-tillering stage while completely covering the old leaves [55] (Figure 1). Wang et al. [55] demonstrated with genetic experiments that the cell death of the *spl3* mutant was caused directly by excess amounts of the H_2_O_2_ accumulation by comparison with that of the normal leaves in wild-type rice. Interestingly, they also reported that the *spl3* mutant was unresponsive to abscisic acid (ABA) with delayed leaf senescence and a loss of catalase activity. This resulted in a failure to scavenge the ROS, specifically H_2_O_2_, causing lesion formation on *spl3* leaves [55]. Furthermore, the *spl3* mutant locus was mapped on the Os03g0160100 and encoded the Mitogen-Activated Protein Kinase Kinase Kinase 1 (OsMAPKKK1) [55]. Thus, the *spl3* mutant can be considered as a genetic model to explain the molecular mechanism for plant stress-signaling hormones, including the MAPK cascades in ethylene signaling, ABA-signaling, and resistance to stress and pathogens in rice.

The rice *spotted leaf 4* (*spl4*) mutant is specifically linked to large reddish-brown spots scattering on leaves [75] (Figure 1). Of note, 40 years after the *spl4* genetic mutant was identified and named the gene *LESION RESEMBLING DISEASE 6-6* (*LRD6-6*), the DNA locus of the gene was found to be the Os06g0130000 locus [62]. Song et al. [62] reported that the gamma ray-induced *spl4*-*1* mutant was caused by a single base substitution in the Os06g0130000 DNA segment, resulting in the production of a nonfunctional ATP-dependent microtubule-interacting-and-transport (MIT) spastin protein. Song et al. [62] also suggested that the *spl4-1* mutant inhibit the polarization of cortical MTs in internode caused by damage in the spastin protein function, which results in a shortened internode; hence, the *spl4-1* mutant allele exhibited semi-dwarfism.

The rice *spotted leaf 5* (*spl5*) mutant displayed reddish-brown lesion spots on leaves from the seedling stage followed by proliferation to the complete leaf surface with maturation (Figure 1). The gene of the *spl5* mutant was cloned as the Os07g0203700 locus, which encodes a splicing factor 3b subunit 3 (SF3b3) and involved in splicing premature RNAs [49,64]. Chen et al. proposed that the SPL5 protein regulates the RNA splicing mediated in the negative regulation of cell death for pathogen resistance [49]. Moreover, a frame shift mutant of the *spl5* gene was noted in the premature termination of its transcription. Thus, the mutant lost regulation of RNA splicing, which results in programmed cell death (PCD) to develop lesions on leaves [49].

Yamanouchi et al. first identified the rice *spotted leaf 7* (*spl7*) mutant, which encodes a heat stress transcription factor and regulates cell death, and a defense mechanism in rice [14]. Of note, the rice *spl7* mutant was located on the Os05g0530400 locus encoding a heat stress transcription factor and regulates the resistance and defense mechanism in the *spl7* mutant [14].

The rice *spotted leaf 11 (spl11)* mutant, cloned on the Os12g0570000 locus encodes the SPL11 protein, shows red spots distributed on the leaves, and confers the non-race-specific resistance to blast and bacterial blight [14]. The wild type of SPL11 protein contains both a U-box domain and an armadillo (ARM) repeat domain [45]. Interestingly, the proteins with U-box and ARM domains were examined as the key components in apoptosis by ubiquitination and protein–protein interactions in yeast and mammalian systems, respectively [76]. In plants, ubiquitination-mediated protein degradation is an important process required in the photomorphogenesis and regulation of hormone signaling [77]. The *spl11* mutant was produced by the single nucleotide substitution of the wild-type SPL11 protein, resulting in nonfunctional protein for the E3 ubiquitin ligase activity for ubiquitination. Therefore, the lesion spots of the *spl11* mutant leaves were triggered by cellular homeostasis failure and an interruption of negative regulation of PCD for defense activation [45]. It is an essential hypothesis in that the E3 ubiquitin ligase activity mediates the ubiquitination system to control the PCD and defense in plants [45].

The rice *spotted leaf 18* (*Spl18*) mutant identified on the Os10g0195600 locus encodes an acyltransferase protein by the T-DNA gene tagging [22]. The *Spl18* showed a dominant phenotype with resistance against disease. Furthermore, plants overexpressing the acyltransferase protein of the *Spl18* gene also showed resistance to bacterial blast disease.

The rice novel *spotted-leaf 28* (*spl28*) mutant was identified by the treatment with N-methyl-N-nitrosourea (MNU) and showed a typical rice *spotted-leaf* phenotype with small lesions in progressing vegetative development [48]. In particular, the MNU-induced *spl28* mutant showed a gain of pathogen resistance against the rice blast and bacterial blight. The gene for the *spl28* allele was designated at the RAP locus Os01g0703600 and encoded a clathrin-associated adaptor protein complex 1 medium subunit μ1 (AP1M1), which is involved in the post-Golgi trafficking pathway [48]. The clathrin protein performs critical roles in forming clathrin-coated vesicles (CCV) in the cytoplasm for intracellular trafficking as a type of cargo at the cell membrane, trans-Golgi network, and endosomal compartments for multiple membrane traffic pathways. Qiao et al. reported that the *spl28* mutant is due to a dysfunction of CCV formation, which results in the failure of vesicular trafficking and, hence, causes the formation of a hypersensitive response (HR) [48]. This is an important finding in that hypersensitivity to ROS is involved in the destruction of vascular transport systems and may be resistant to pathogens.

The rice *spotted-leaf 30* (*spl30*) mutant, a phenotypic mutant caused by a single recessive gene, exhibits red-brown lesions in response to the light and temperature [78]. Ruan et al. confirmed that the *SPL30* gene located at the LOC_Os12g37870 encodes the ATP-citrate lyases A2 (OsACL-A2) protein [65]. Specifically, the rice *spl30-1* mutant was caused by the defective function of the OsACL-A2 protein. Overall, the rice *spl30* gene is positioned at the Os12g0566300 locus of RAP [65]. The cell death spots of the rice *spl30-1* mutant were identified as a homozygous recessive allele caused by the OsACL-A2 enzyme dysfunction, which results in the significant accumulation of ROS and, hence, accelerates the degradation of nuclear deoxyribonucleic acids [65].

The rice *spotted leaf 32* (*spl32*) mutant, caused by a recessive allele, showed small lesion spots on the leaves from the seedling stage and covered longitudinal lesion strips and leaf veins with the development process. The normal *SPL32* gene was cloned on the Os07g0658400 locus encoding a ferredoxin-dependent glutamate synthase (Fd-GOGAT) (Table 1) [66]. Therefore, the necrosis lesion of the rice *spl32* mutant resulted from the null function of the Fd-GOGAT protein by a single base mutation in the open reading frame (ORF) of the Fd-GOGAT gene [66].

The rice *spotted leaf 33* (*spl33*) mutant showed lesion spots on the leaves caused by ROS accumulation and the PCD-mediated cell death resulting in early leaf senescence. The *spl33* gene was located at the Os01g0116600 locus and noted for the production of eukaryotic translation elongation factor 1 alpha (eEF1A)-like protein composed of a nonfunctional zinc finger domain and three functional EF-Tu domains [67]. Based on the expression analysis of the wild-type *SPL33* gene, Wang et al. proposed that an eEF1A-like protein mediates the PCD and provides resistance against pathogens [67].

The rice *spotted leaf 40* (*spl40*) mutant showed lesion formation at the leaf tips during the seedling stage, which progressively dispersed over the whole leaf surface at the tillering stage depending on the light influx and demonstrated enhance bacterial blight resistance in rice [69]. The gene of *spl40* was defined as the Os05g0312000, but it has not been characterized in rice completely.

The *light-induced lesion mimic mutant (LIL1)* of rice exhibited the light-induced, red-colored lesions on the leaf blades during the development stages. The lesion formation of the *LIL1* mutant was carried out by a semi-dominant allele based on genetic analysis [17]. Recently, the *LIL1* gene was identified on the Os07g0488400 locus and encodes a putative cysteine-rich receptor-like kinase (CRK), predicted based on a Map-based cloning technique [17]. Further research showed that the *LIL1* mutant phenotype was caused by a base substitution mutation in the fourth exon of the LOC_Os07g30510 (MSU ID) [17].

The rice *bilateral blade senescence 1* (*bbs1*) showed early leaf senescence after the ethyl methane sulfonate (EMS) treatment. The leaves of the phenotype *bbs1* were distinguished by the bilateral blade margins with the withered and yellow-colored and governed by a single recessive nuclear gene [50]. The normal *OsBBS1* gene locus was cloned as the LOC_Os03g24930 (MSU) and the Os03g0364400, which encoded a receptor-like cytoplasmic kinase 109 (RLCK109) predicted by the map-based gene cloning method [50]. The mutant phenotype *bbs1* was caused by guanine (G) insertion mutation in the ORF, resulting in a frameshift mutation [50].

A lesion mimic mutant *lmm24* was isolated from ZhongHui8015 (ZH8015) rice after ethyl methane sulfonate (EMS) treatment [16]. The *lmm24* exhibited spontaneous cell death from the seedling stage to the yellow mature stage and enhanced resistance to rice blast fungus *M. oryzae* [16]. Zhang reported that the *lmm24* was caused by a deletion mutation of the LOC_Os03g24930 (MSU) sequences for the RLCK109. Therefore, both the *llm24* and *bbs1* mutants were elucidated to be the result of mutations in the same gene encoding RLCK109 protein. Furthermore, both independent findings showed the receptor-like cytoplasmic kinase 109 (RLCK109) involvement in the immune signaling pathway for the defense response [16].

The rice *white stripe leaf/panicle* (*wsp1*) mutant showed green and white sectors distributed along the major veins in the leaves by a deficiency in chloroplast development [70]. The *WSP1* gene was located on the Os04g51280 locus and encoded a putative product of the multiple organelle RNA editing factor 2b (MORF) proteins. The white striped leaf phenotype of the *wsp1* mutant was caused by a nonfunctional MORF protein to regulate inaccuracies in RNA editing in chloroplast development. Zhang et al. reported that the WSP1 factor is essentially required for chloroplast development in rice [70], contributed to understand the chloroplast development and function.

The rice *spotted leaf sheath* (*sles*) mutant showed lesion mimic spots on the leaf sheath with early senescence and was controlled by a single recessive nuclear gene [79]. Lee et al. reported that the *sles* mutant phenotype varied from the rice spotted leaf mutants [79]. The lesion spots of the *sles* mutant appeared on the leaf sheath at the two-leaf stage and later expanded to cover the entire leaf sheath rather than on the surface of leaf. Genetic mapping showed that the *sles* mutant was located at the LOC_Os07g25680, which encodes a kinase domain-containing protein of the Raf MAPKKK family. 

In the *sles* mutant, ROS scavenging genes and pathogenesis-related (PR) genes were upregulated significantly. The discovery of the *sles* mutant reaffirmed that Mitogen-Activated Protein Kinase Kinase Kinase (MAPKKK) is involved in the regulation of ROS homeostasis. Therefore, the failure of the MAPK cascade functions in the *sles* mutants results in unbalanced ROS homeostasis, leading to cell death and accelerated senescence by ROS accumulation in the leaf and sheath [79]. Thus, understanding the genetic mechanism of the *sles* mutation linked with ROS homeostatic regulation may further provide clues on the pathogenic resistance of rice.

In molecular genetic studies of rice LMM mutations, the lesion formations in LMM rice were caused by the abnormal ROS production in the cell physiology or dysfunctional proteins for ROS scavenging systems [69]. Based on the Mendel’s inheritance, most LMMs were caused by recessive alleles; hence, several LMMs were mapped for the DNA locus and proteins in rice genomes (Table 1). Therefore, the interpretation of genetic mechanisms in rice LMMs can be used to define the PCD and resistance to diseases.

## 3. Development of Lesion Formation in Rice LMMs

Rice and maize showed similar types of lesion formation and are modulated during the development process [19,27]. Although no spots were reported in newly formed young leaves, the metabolically active flag leaves showed severe lesions [19,21,27]. In most cases, initially, lesions first appeared as specks near the tip of the leaf and then eventually, completely covered the leaf. Finally, the leaf surfaces exhibited yellow, brown, red, and black spots of dead necrotic cells [21] (Figure 1).

Many studies reported that lesion formation accelerated the severity of spot formation in rice leaves under high temperature and high light intensity [16,24,78]. Depending on the developmental differences of the lesion formations, the LMM phenotypes could be categorized into whole life lesion mimics, vegetative initiation lesion mimics, and reproductive initiation lesion mimics [7,8,26,28,80,81]. There are significant differences in the patterns of lesion formation of the LMM genes, particularly the spot color, size, and intensity (Figure 1). Most lesion colors of LMMs typically present yellow, brown, and red spots. However, the *white-stripe leaves and panicles 1* (*wsp1*) mutant showed white-stripe leaves and panicles in rice [70]. 

Recently, a novel *white-spotted leaf* mutant was also identified in a japonica-type Korean cultivar that showed both brown spots and white lesion spots on the tip of the leaves from the vegetative stage [40]. In the *spl5* mutant, the spot intensity was less than the others, whereas *bl1, bl2, spl3, spl4*, and *spl6* produced a dense intensity of spots (Figure 1). Thus, various spot sizes, colors, and patterns in LMMs indicate the involvement of different genes. For example, the spot number and spot intensity were inversely proportional, where a few spots were noted with large spot size as found in *bl2* and *spl4* (Figure 1).

The formation of spots on most rice *spl* mutants of LMMs was controlled by the developmental pattern, as it was formed at a certain stage of plant age. Once spots are developed on all the leaves, they mostly remain in all the developmental stages. Interestingly, the flag leaves are affected severely by the lesion spots resulting in earlier senescence. In most *spl* mutants, no visible spots existed on the leaf at the seedling stage; however, spots were appeared as tiny spots at the tillering stage (45 days) and developed into severe phenotype at the milk stage (60 days) under natural field conditions between 30 to 35 °C [21]. 

Lesion formation can spread throughout the generations where the lesions initiate sparsely, and once formed, they can expand quickly over the entire leaf blade. Most of the spots were formed throughout the minor leaf veins and rarely on the central vein. Finally, lesions were spread all over the leaf surface that caused earlier leaf senescence and the death of the localized cells (Figure 1). Except for leaf blades, no other organs were affected by premature cell death because no visible spots existed, indicated that the photosynthetic organ produce the spots due to the accumulation of high ROS levels during photosynthesis and results in lesion formation by cell death.

In addition, the leaf portions of the *spl6* plants protected from sunlight showed resistance to the development of lesions, it indicated the influence of light via photosynthesis in the induction of the spot formation on the mutant leaves. Similarly, researchers reported that fewer lesions were formed on leaves covered with aluminum foil [82,83]. To note, the covered area on the leaf either did not develop any spots or fewer lesions were formed by comparison to that of the non-covered area [72]. Interestingly, the lesions development was noted only under white light, while no lesion formation and reduced lesion formation were documented on the leaves exposed to either blue or red light [26,84]. Hence, the lesion formation of LMMs may be linked directly to the light reactions of the photosynthesis process.

Some patterns of lesion spot formation in rice LMMs are classified as progressive types during the developmental stages, including *spl3, spl4*, and *spl6* mutants [14,19]. Typically, these mutants are arranged in groups of longitudinal lines along the leaf blade as dense spots towards the leaf apex rather than the leaf base (Figure 1). Noticeably, rice *spl* mutants produced more than two times number of spots with an increase in temperature, which was similar to *Lls1* and *slm1* mutants in maize [20,82,83]. 

**Figure 2:**
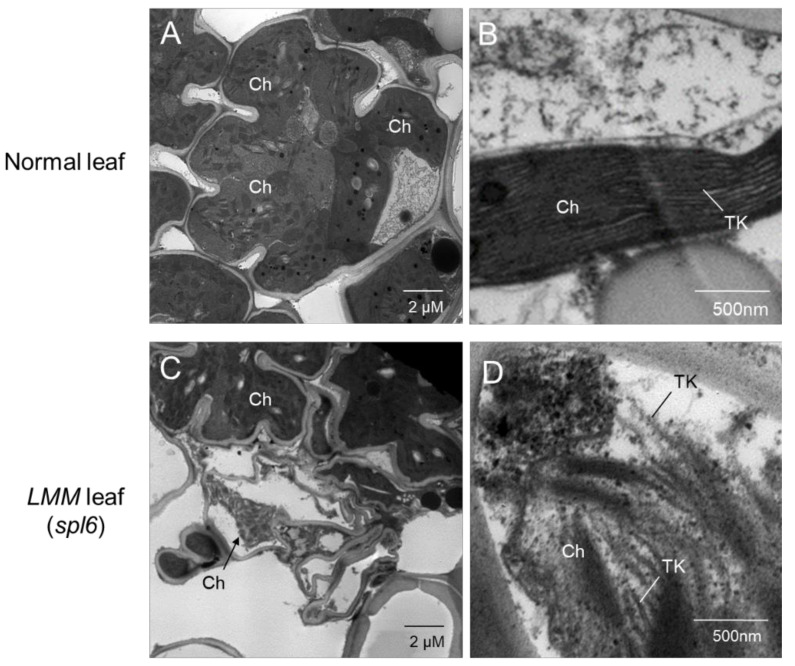
Electron microscopy images of *spl6* and normal wild-type rice leaves, (**A**,**B**) Dense chloroplasts of normal wild type and (**C**,**D**) degraded chloroplasts (Ch) in the *spl6* LMM mutant. Normal leaves of wild type showed that chloroplasts were well-developed in mesophyll cells with a firm structure of thylakoids (TK), but typical LMM leaves (*spl6*) showed the progression of chloroplast degradation in some mesophyll cells with broken lamella and thylakoids (arrows).

In the *spl6* mutant leaves, the highest levels of H_2_O_2_ accumulation were detected, which caused the initiation of severe cell death at a plant age of 45 days, suggesting the toxic accumulation of H_2_O_2_ in plants to initiate the cell death [85]. Such a fatal process was observed, particularly when the developing lesions of *spl6* were closely dissected and the thylakoid membranes in the chloroplast of the spotted leaves were damaged severely (Figure 2). 

This is because the lesion-formed cells failed to remove excessive amounts of ROS with the progression of photosynthesis. Moreover, the light intensity and temperature, which enhance the photosynthesis rate, were also marked as critical environmental factors that trigger the phenotypic expression of lesions [9,11,12,14,20]. For example, the rice *spl7* mutant showed a variable lesion density under various temperature conditions, where the leaves showed a reduction in lesion density under low temperatures [14]. Whereas the *spl6* mutant exhibited an increase in lesion spot formation corresponding to high light intensity [20].

Furthermore, Yamanouchi et al. proposed that the lesion formation of rice *spl7* mutants was caused by the dysfunction of a heat stress transcription factor (HSF) [14]. As reviewed, different phenotypic expression pathways of the LMMs may exist specifically that depend on the genotypes of LMMs (Table 1). On the other hand, due to genetic differences, the *spl6* gene is located on chromosome 1, and the *spl7* gene is on chromosome 5; thus, the *spl6* mutant can be caused by a functional protein other than HSF of the *spl7* mutant. Moreover, the *brown leaf* (*bl*) mutant and *spl* mutants showed varied lesion patterns on the leaves. Hence, the LMMs may differ in each gene mutation; however, the lesion formation in similar phenotypes occurred due to the failure of scavenging excess ROS by various genes.

## 4. Chloroplast Damage from Disrupted Photosynthetic Apparatus in LMMs

During lesion formation in the LMMs, the chloroplast is the primary target to be damaged, which affects the photosynthetic machinery. The rice LMM traits cannot control the level of photosynthesis, resulting in plant death. Chlorophyll is a photosynthetic pigment that is positively correlated with the photosynthetic rate [86]. The chlorophyll content usually increases with increasing plant age. 

On the other hand, the chlorophyll content decreased slightly in the rice later during senescence [87]. In the presence of light, chloroplasts in plant cells are the major source of ROS and have been shown to control the PCD in response to reactive oxygen species [88]. However, no significant differences in the chlorophyll content were noted in the later stages of wild type plants, while substantial differences were observed in the lesion spots of the *spl6* mutant, predicted as a result of damage in the lamella and thylakoid membrane of chloroplasts (Figure 1 and Figure 2) [20].

The genetic basis of chlorophyll is quite complex, and many metabolic steps and genes are involved [87,89]. The *RuBisCO* is abundant mostly in the normal chloroplasts, indicated a strong correlation of *spl* mutant with reduction in *RuBisCO* at a severe cell death stage [19]. In the *spl6* mutants, the induction of ROS, which causes reduced transcription level and cell death, is marked for a decrease in chloroplast production and reduced photosynthesis. 

As depicted in Figure 2, TEM images show heavily damaged chloroplasts or an absence of mesophyll chloroplasts in the *spl6* mutant by comparison to wild-type. Damaged thylakoid membranes in the mesophyll chloroplasts were also observed in the non-spotted area of the *spl6* mutant, and no chloroplasts were observed in the mesophyll cells from the spotted area of the *spl6* mutant. Chloroplast damage was caused by an oxidative burst, which results in excessive deposition of substances, such as plastoglobules and callose, in chloroplasts [20,24]. This suggests that overexpression of the ROS is linked to lesion formation and cell death in the LMMs, facilitating the mechanisms underlying protection against stress conditions. 

Similar to the *spl6*, the degradation and changes in the chloroplast structure were also observed in the *spl36* [24]. Moreover, the rice *spl26* mutant was observed to disturb the photosynthetic capacity by a reduction in chlorophyll content accompanied by an increase in the membrane ion leakage rate and malonaldehyde level [90]. As another example, the *spl33* mutant impaired the downregulation of photosynthesis-related genes and the up-regulation of senescence-associated genes. 

In addition, it also showed a loss in chlorophyll and a breakdown of chloroplasts [67]. The rice *llm1I* mutant showed chloroplasts damaged by a malfunction of the cysteine-rich receptor-like kinase [55]. In summary, the genetic analysis of rice LMM traits showed that an uncontrolled photosynthesis rate resulted in the formation of lesions, which causes phytotoxicity in the plant.

## 5. Chlorophyll Content and Lesion Severity

Defects in the chlorophyll catabolism can also cause cell death in plants. A disruption of two essential enzymes, i.e., pheophorbide a oxygenase and red chlorophyll catabolite reductase, are involved in the degradation of chlorophyll, which generates spontaneous lesion in the *accelerated cell death 1 (acd1)* and *accelerated cell death 2* (*acd2*) mutants [91,92,93]. Also, *Arabidopsis acd1* was characterized by the accumulation of pheophorbide a (pheide a) due to the disruption of the *ACD1/LLS1/PHEIDE A OYGENASE (PAO1)* gene [7,93]. Of note, the primary function of the pheophorbide a oxygenase is to convert the pheide into the red chlorophyll catabolite (RCC) [94,95]. However, excessive accumulation of the photoreactive pheide in the chloroplast induces cell death in a light-dependent manner [96]. In addition, the *ACD2* gene encodes an RCC reductase, which catalyzes the subsequent reaction and converts the RCC into the primary fluorescent chlorophyll catabolite [92]. Thus, the spontaneous cell death observed in the *acd2* mutant was correlated with the accumulation of the RCC and singlet oxygen (^1^O_2_) formation [95].

Moreover, the ACD2 may bind to a porphyrin-related molecule in the mitochondria to prevent the PCD [97]. Interestingly, during pathogen infection, the RCC reductase as product of the *ACD2* gene dynamically localizes between the chloroplast and mitochondria, resulting in protection against mitochondrial oxidative bursts [98]. Many LMMs are affected by chlorophyll biosynthesis or degradation; it is unclear whether a change in the accumulation of photoreactive chlorophyll precursors or degradation product contributes to the PCD. Chloroplasts play a key role as a sensor of light stress that delays the photosynthetic electron transport and regulates the ROS production. 

Among the ROS, singlet oxygen (^1^O_2_) specie activates a signaling pathway in chloroplasts that is regulated by the two plastid-localized proteins, EXECUTER 1 and 2 [99]. The EXECUTER (EX) proteins exhibit protective role in the wild-type plant under high light stress conditions [100]. Therefore, the EXECUTER 1 activation assists to halt the stress by the release of singlet oxygen.

The ROS is generated in the chloroplast from various pathways that further triggers an HR and initiate the appearance of light-dependent LMM phenotypes owing to the failure of the plant machinery to dissolve excess excitation energy (EEE) [101,102,103]. This is highlighted by the light-dependent LMMs, including *lsd1, acd1*, and *acd2*, which showed defects in the EEE dissipation or chlorophyll catabolism caused by photooxidative damage and the formation of the ROS [92,94,104,105]. In addition, the light-induced *bl2* and *spl6* mutants progressed the destruction of thylakoids in chloroplasts, resulting in lesion formation (Figure 2) [21,85]. Hence, collected evidences suggest that the lesion formation of light-induced LMMs may be caused by genetic defects in the EEE distributions machinery of the chloroplast.

## 6. Reactive Oxygen Species Cause Lesions by in the PCD of the LMMs

**Figure 3:**
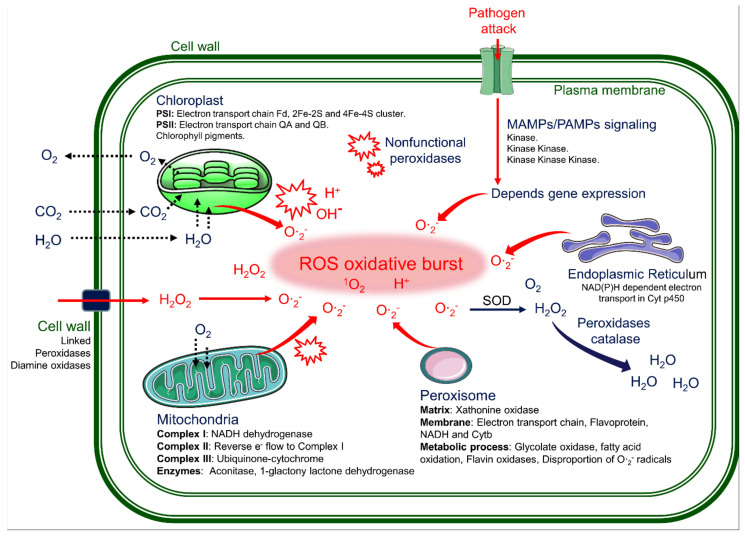
Schematic diagram for the sites of the ROS production from enzymatic and non-enzymatic complexes during an “oxidative burst” under biotic and abiotic stress. Also, as a result of the defense mechanism of pathogen infections, an “oxidative burst” occurs, which is associated with microbe/pathogen-associated molecular patterns (MAMPs/PAMPs) for identification of attacking pathogen. Oxidative burst mechanisms in the cellular response are different in innate genetic LMM phenotypes and pathogen attack; however, the results are similar. SOD, Superoxide dismutase.

**Figure 4:**
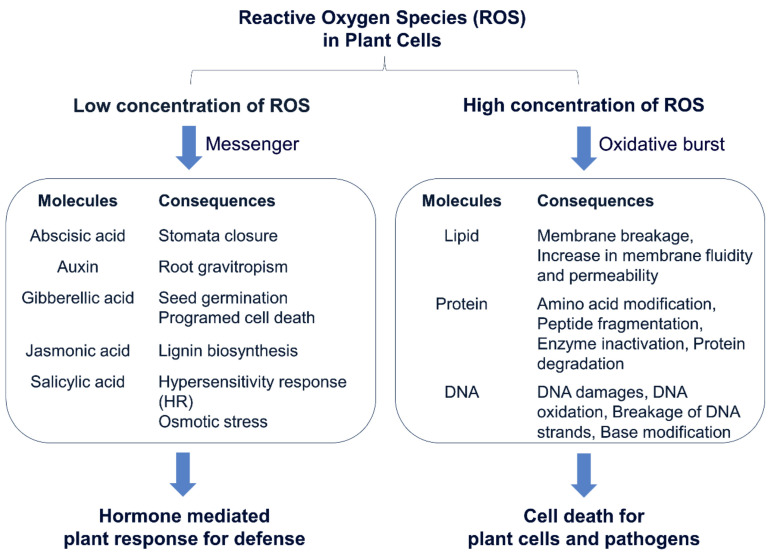
Summary on the role of the ROS at low and high concentrations for the plant and pathogen. The ROS at low concentrations acted as a second messengers in several plant responses, including stomatal closure, root gravitropism, seed germination, programmed cell death, lignin biosynthesis, the hypersensitivity response, and osmotic stress in plant cells for defense. However, high concentrations of the ROS induce cell death through oxidative damage to the lipids, proteins, and DNA in plant cells and pathogens.

The extensive formation of lesions in rice LMMs are induced by abiotic stresses, such as water, temperature, and light, and biotic stresses, such as reactive oxygen species (ROS) and pathogen infection [19,21,82,106,107]. In particular, the ROS can partially reduce or activate the derivatives of oxygen, such as superoxide radicals (O∙_2_^-^), hydroxyl radicals (OH∙), singlet oxygen (^1^O_2_), and hydrogen peroxide (H_2_O_2_). The ROS are always formed by the inevitable leakage of electrons to O_2_ from the electron transport activities of chloroplast, mitochondria, and plasma membranes or as a byproduct of various metabolic pathways localized in different cellular compartments [108]. Therefore, these ROS molecules are highly reactive and toxic and lead to the oxidative destruction of cells. A large amount of the ROS leads to the destruction of several subcellular organelles, such as chloroplasts, mitochondria, the plasma membrane, peroxisomes, apoplasts, the endoplasmic reticulum, and the cell wall in rice LMMs (Figure 3 and Figure 4).

Most of the lesion formation processes in LMM plants are rapid and cause the spontaneous death of localized cells at the location of stress. Physiological evidence in LMM studies concluded that a sudden increase in the ROS production in the mesophyll cells of a spotted leaf is a causal agent for cell death. For example, high accumulations of the H_2_O_2_ and superoxide (O_2_^−^) in the lesion area of spotted leaves have been identified in *spotted leaf* mutants [8,10,17,55,85]. 

During rapid cell death caused by lesion formation in leaves, several toxic substances have been found to delay the electron transport chains (ETC) and to block antioxidant enzymes, leading to the excessive accumulation of the ROS. This accumulated ROS finally results in oxidative damage, lipid peroxidation, membrane leakage, and localized cell death [21,109] (Figure 2 and Figure 3). The destruction of the thylakoid membrane has been shown in images of chloroplasts in mesophyll cells in situ (Figure 2) [20,21]. Therefore, the ROS eruption is the main cause of lesion spot initiation.

The normal amount of the ROS in cells has been implicated as secondary messengers in intracellular signaling cascades that mediate several plant responses in plant cells, including stomatal closure at low or moderate concentrations [110], programmed cell death [111], root gravitropism [112], and the acquisition of tolerance to both abiotic and biotic stresses [113]. In addition, the low concentration of the ROS served as a messenger to induce the ascorbic acid production, which constitutes an essential substance in the network of antioxidants. In the *spl6* mutant, the ascorbic acid contents in lesion-developed leaves showed gradual increment with an increase in the spot severity (Figure 4) [20].

The occurrence of the ROS is inevitable due to plant photosynthesis. On the other hand, the ROS elimination in plant cells is achieved by the ROS scavenging system, which is mediated by various enzymatic defense systems, including superoxide dismutase (SOD), catalase (CAT), and peroxidases (POX) [114]. The SOD catalyzes the superoxide into oxygen and H_2_O_2_ [115]. Also the CAT plays a role in the catalysis of the H_2_O_2_ in cells [116], while the peroxidases (POD) catalyze the oxidation of phenolic compounds and reduce the H_2_O_2_ production [101].

Under high ROS levels, the H_2_O_2_ is generated endogenously by the metabolic pathway and induces the PCD in plant cells [117]. The H_2_O_2_ is reduced by the protein thioredoxin peroxidase (TPX) through the intracellular redox signaling pathway [118,119]. On the other hand, the reduced level of the TPX in the LMMs failed to eliminate the H_2_O_2_, leading to the PCD and consecutive lesions in the spotted leaves of the *spl1* and *spl6* mutants [20,59,120]. 

Therefore, ROS accumulation in LMM plants may cause the extensive lesions formation mediated by PCD, resulting in a broad spectrum tolerance to the stress responses and pathogens [121,122]. Additionally, the ROS accumulation in LMMs also affects plant growth and differentiation.

## 7. Role of the MAP-Kinases Pathway in the PCD of the LMMs

In pathogen attack, the PCD is processed rapidly to protect plants, and several protein kinases play important roles in regulating the stress signal transduction pathways (Figure 3). During biotic and abiotic stresses, several specific kinase-signaling pathways should be considered to initiate an oxidative burst in the LMMs. This evidence was reviewed in several LMMs with a typical phenotype of the PCD without a pathogen attack. Besides, the mitogen-activated protein kinase (MAPK) cascade plays an important function during external environmental stimuli, thereby performing the transduction of extracellular signals to intracellular targets [123]. 

Recently, the MAPKKK cascade was also discovered to cause lesion spot formation in rice [55]. The protein of the *spotted leaf sheath* (*sles*) gene was also discovered to contain Raf MAPKKK [79]. Thus, the nonfunctional Raf MAPKKK of the *sles* mutant may be associated with loss of the ROS homeostatic regulation, which results in the oxidative burst for lesion formation in leaves. The MAPK cascade contains many other kinases, including MAPKs, MAPKKs (MAPK kinases), and MAPKK kinases (MAPKKKs) [124,125,126]. Plants have more MAPKKKs than MAPKs and MAPKKs, which leads to complicated and fickle regulatory MAPK cascades. On the other hand, some MAPKKKs have been characterized and are involved in several biological processes, including biotic [127,128] and abiotic stress responses [129,130], innate immunity [131], and the defense and stress responses [121]. Conclusively, the MAPK cascade function is critical to modulate the ROS homeostasis in plants; hence, it needs to be investigated for genetic mechanisms linked with oxidative bursts in the LMMs.

## 8. Role of Sphingolipids in LMMs

Sphingolipids are ubiquitous in a wide variety of organisms, including eukaryotes and prokaryotes. Ceramides, derivatives of sphingolipids, are components of the cell membrane and participate in various cellular signaling processes. Examples include regulating differentiation, proliferation, and programmed cell death (PCD) [132]. In plant-microbial interactions, pathogen-specific sphingolipids were found to induce the Ca^2+^ signaling, MAPK, and ROS production in rice [133]. 

Genetic evidence has shown the ceramide functions in plant PCD. For example, the *ACD5* (*ACCELERATED CELL DEATH5*) and *ACD11* encoded genes for the ceramide kinase and sphingosine transfer proteins, respectively, are involved in the sphingolipid metabolism [134]. The *acd5* mutant helped to reduce the ceramide kinase activity and displayed an enhanced cell death after bacterial pathogen infections. The reduction of ceramide kinase resulted in the accumulation of precursor molecules, likely ceramide or sphinganine [135]. 

Therefore, the homeostatic balance between ceramides/sphingolipids and phosphorylated derivatives is important for lesion formation and cell death in plants. Another study showed that the *acd11* lesion mimics a mutant of *A. thaliana* that exhibits an autoimmune phenotype, such as constitutive defense responses and death without pathogen infections [117]. *ACD11* encoded a putative sphingosine transfer protein; however, the precise role during these processes is still unknown.

On the other hand, the defeat of ceramide kinase in the *acd5* mutant of *Arabidopsis* sp. lost defenses to the ceramide accumulation and mitochondrial H_2_O_2_ bursts [136]. The physiological effects of the PCD mediated by the sphingolipid metabolism found in *Arabidopsis* LMMs are similar to rice LMMs; however, the underlying mechanism related to ceramide functions is not well known in rice LMMs.

## 9. Resistant to Pathogen Infection in the LMMs

Upon pathogen attack, the ROS is induced immediately to kill the infected cells and served as a signal to activate the defense response [137]. The ROS studies with findings in LMMs showed that low ROS concentrations acted as a second messenger in several plant responses, including stomatal closure, root gravitropism, seed germination, programmed cell death, lignin biosynthesis, hypersensitivity responses, and osmotic stress (Figure 4). Upon stress induction, the accumulation of salicylic acid was observed, which leads to an increase of the endogenous level of H_2_O_2_, which could then serve as a secondary messenger to induce the PCD. 

This ROS-mediated lesion formation of the LMMs may induce the systematic resistant system against pathogens. To avoid oxidative damage from the ROS, the antioxidant enzymes, including SOD, CAT, APX (Ascorbate peroxidases), and GST (glutathione-s-transferase), help to remove excess amounts of the ROS. Enzymatic defenses include superoxide dismutase, which converts the superoxide radical to the H_2_O_2_ while the catalases and peroxidases, which trigger the conversion of H_2_O_2_ to water and oxygen under normal conditions and can handle the oxidative load [138]. In this way, most LMMs may show enhanced resistance to rice blast and bacterial blight [13,17,22,23,24,48,62,69,106,138,139,140,141]. Moreover, the ROS accumulation due to the loss-of-function of the ROS scavenging systems in the LMMs affect the respiratory burst oxidase homolog (Rbohs)-mediated signaling in plant growth, differentiation, and reactions to biotic and abiotic stresses [141,142,143]. Thus, it is predicted that some of the rice *Rboh* gene families in LMMs may contribute to resistance against pathogens.

*SPL35* was shown to enhance the disease resistance to fungal and bacterial pathogens [68]. Furthermore, the *bbs1* (*lmm24*) mutant showed up-regulation of defense response genes and resisted the rice blast fungus *Magnaporthe oryzae* [16]. For example, the excessive accumulation of the H_2_O_2_ in the leaves of the *spl5* mutant induced the HR and increased the resistance to pathogens [49,55]. Thus, the up-regulation of the APX and GST are hypothesized to act as scavenger of the ROS in the *spl5* mutant. However, experimental observations showed insufficient production of the APX and GST in the *spl5* mutant to detoxify the overproduction of the ROS during plant growth.

In rice, failure of the vesicular trafficking system is involved in cell death. Rice *AAA-ATPase1* plays a role in the association with salicylic acid-regulation for disease resistance and defense responses against the blast fungus *M. oryzae* [63]. In another study, the *lrd6-6* mutant showed multivesicular bodies (MVBs)/endosomes-mediated vesicular trafficking, which might play important roles in plant immunity and cell death [61]. Similarly, the *spl28* mutant failed to exhibit normal vesicular trafficking resulting in spotted leaves and early senescence. On the other hand, the LMMs can act as a negative regulator of cell death with enhanced resistance to rice blast and bacterial blight [60].

Recent studies reported that rice LMMs would be helpful in resistant breeding because most of them showed spontaneous activation of defense responsive genes in pathogen attacks [15]. Manigbas et al. reported that UV radiations could induce an increase in the H_2_O_2_ production, which could inflict injury to crops affecting growth and productivity [142]. Therefore, some rice LMM plants were selected as the rice breeding lines related to abiotic stresses, such as UV and H_2_O_2_ resistance. 

In particular, catalase (CAT) in the *spl6* and *bl2* mutants was upregulated during lesion development, resulting in enhanced ROS scavenging in damaged cells, thereby, lowering the H_2_O_2_ levels [20,142]. For example, the *SL* gene of the *Sekiguchi lesion* (*sl*) mutant encoding a P450 monooxygenase protein-producing serotonin showed pathogen defense responses for rice blast disease [59,73,74]. Therefore, most LMMs have resistance to pathogenic infections depending on their genes, which are useful in genetic resources breeding disease-resistant plants.

## 10. Conclusions and Future Prospects

The plant lesion mimic mutants (LMMs) show aberrant regulation of cell death without a pathogenic infection. Genetic analysis of the rice spotted leaf genes of the LMMs showed that a recessive gene governs most LMMs. This study reviewed rice LMMs regarding the morpho-physiological features, biochemical functions, subcellular localization, and protein interactions. Most of the rice LMMs failed to scavenge the excess ROS and excitation energy generated during photosynthesis. Therefore, the ROS burst resulted in the formation of lesion spots mediated by the PCD. Some rice LMMs exhibited enhanced disease resistance via the consecutive production of the ROS, which can provide insight into the mechanisms underlying the HR, PCD, and immunity.

The lesion phenotypes and the genetic mode of rice LLMs are also involved in the chlorophyll metabolisms, stress tolerance, and ROS detoxification pathways. Consequently, more intensive research will be needed to obtain further insights, identify the target molecules, explore more mutants, and obtain genetic definitions of the corresponding genes. 

The necrotic lesion spots caused by the ROS burst induce cell death for both the pathogens and plant cells to prevent the spread of invading pathogens throughout the plant body. Therefore, the rice LMM genetic traits are commonly elucidated to have highly developed self-defense mechanisms. Moreover, the identified LMM genes exhibiting the molecular genetic mechanisms of lesion formation will be useful for breeding rice with disease resistance and environmental stress tolerance as well as to achieve a high yield with improved photosynthesis efficiency.

## Data Availability

The data of this article is within the text.
